# CRISPR-targeted microbiome modulation in rare Henoch-Schonlein purpura IgA nephropathy-gastritis: insights into the gut-kidney axis

**DOI:** 10.1097/MS9.0000000000004466

**Published:** 2025-12-03

**Authors:** Pakeeza Saif, Muhammad Tausif, Mahnoor Fatima, Abdullah Imtiaz, Muammar Hassan

**Affiliations:** a Department of Medicine, King Edward Medical University, Lahore, Pakistan; b Department of Medicine, Mayo Hospital, Lahore, Pakistan; c Department of Medicine, Shaheed Ziaur Rahman Medical College, Rajshahi Medical University, Bangladesh; d Department of Medicine, Wah Medical College, Wah Cantt, Pakistan

*To the Editor,*

Henoch-Schonlein purpura (HSP), most notably HSP nephritis (HSPN), is an autoimmune vasculitis that primarily targets small vessels in the skin, joints, gastrointestinal tract, and kidneys, causing significant issues in pediatric patients because of the risk of progression into chronic kidney disease (CKD). Recent mucosal biopsies show the deposition of galactose-deficient Immunoglobulin A1 (Gd-IgA1) complexes in the gastric mucosa, which strengthens the gut mucosa as a site of immune dysregulation contributing to systemic mucosal flare-ups and renal damage in HSPN-IgA Nephropathy (IgAN) overlap syndromes^[1]^. The gut-kidney axis is currently being acknowledged as a critical therapeutic target to inhibit ongoing vasculitic progression and to preserve renal function^[2]^. This letter is in line with the TITAN Guidelines on the need for transparency in AI use in health care^[3]^.

Aberrant mucosal immune homeostasis augments the production of aberrantly glycosylated IgA, especially an increase in Gd-IgA1, which forms circulating immune complexes that deposit within the renal mesangium, leading to inflammation and causing the pathogenic link between gut microbiota dysbiosis and IgAN. The altered intestinal barrier and dysregulated mucosal B-cell responses due to dysbiosis are involved in this process^[4]^. Currently, available standard therapies have included immunosuppression, but there is increasing evidence to promote microbiome-targeted approaches^[2]^. Most importantly, there is significant potential for using CRISPR gene editing to alter the composition and functioning of gut microbiota^[5]^. In this context, engineered *Akkermansia* species have been proposed to remodel glycans into the mucosal habitat, mitigating purpuric flares by restoring barrier integrity and reducing pathogenic IgA complex formation^[1,4,5]^.

Arthralgia that occurs in HSP or IgA vasculitis often mimics symptoms seen in inflammatory bowel disease (IBD), complicating clinical differentiation^[6]^. Thus, serum Gd-IgA1 has been proposed as a cross-organ biomarker reflecting shared immunopathological pathways, allowing earlier identification of systemic involvement and tailoring strategies for intervention. The importance of this particular biomarker is underscored in pediatric vasculitis, where being able to recognize and treat it promptly may prevent progression toward irreversible renal damage^[4]^. The introduction of CRISPR-modulated microbiome therapy through biomarker-led clinical monitoring could create a new paradigm for managing such rare but significant diseases^[7]^.

This discussion provides a fresh perspective in the rapidly maturing domain by combining current findings on CRISPR-targeted microbiome interventions, particularly with respect to genetically engineered strains of *Akkermansia*, for an emerging therapeutic target in the rare overlap syndromes of HSP and IgAN-gastritis^[5]^. By putting emphasis on mucosal Gd-IgA1 complexes in gastric biopsies as a measure of localized immune dysregulation beyond renal pathology, it has helped to expand the current knowledge of disease mechanisms^[1]^. Furthermore, the identification of Gd-IgA1 in serum as a prospective systemic biomarker linking together mucosal and joint involvement lends credence to its relevance in gut-kidney axis function as a prime candidate for intervention in pediatric patients^[8]^.

To conclude, the association between the gut–kidney axis and both HSPN and IgAN is increasingly evident. Gastric mucosal biopsies demonstrating Gd-IgA1 complexes highlight that disease involvement extends beyond the kidneys to other mucosal systems. Emerging microbiome strategies, such as the use of genetically modified strains of *Akkermansia* through CRISPR-targeted remodeling of glycan structures, hold promise for mitigating purpura. Early detection of Gd-IgA1 may allow recognition of multi-organ involvement at an early stage, enabling differentiation of disease-related arthralgias from IBD and facilitating timely treatment initiation. Incorporating microbiome-targeted therapies with definitive biomarkers to monitor disease progression could significantly reduce the risk of CKD, making this a priority within the pediatric vasculitis research agenda.

## Data Availability

Not applicable to this study.
